# Association of Dental Caries and Anthropometric Measures among Primary School Children

**DOI:** 10.3390/children8030223

**Published:** 2021-03-13

**Authors:** Wajiha Anzar, Ambrina Qureshi, Ashar Afaq, Hiba F. Kattan, Basil Almutairi, Khaled M. Alzahrani, Mustafa Naseem, Fahim Vohra, Tariq Abduljabbar

**Affiliations:** 1 Department of Community and Preventive Dentistry, Dow International Dental College, Dow University of Health Sciences, Karachi 74200, Pakistan; wajiha.anzar1@gmail.com (W.A.); ashar.afaq@duhs.edu.pk (A.A.); Mustafa.naseem@duhs.edu.pk (M.N.); 2 Department of Community and Preventive Dentistry, Dr. Ishrat-ul-Ebad Institute of Oral Health Sciences, Dow University of Health Sciences, Karachi 74200, Pakistan; ambrinaqureshi@duhs.edu.pk; 3 Preventive Dental Science Department, Princess Nourah Bint Abdulrahman University, Riyadh 11671, Saudi Arabia; hfkattan@pnu.edu.sa; 4 Department of Restorative Dentistry, Division of Operative Dentistry, College of Dentistry, King Saud University, Riyadh 60169, Saudi Arabia; balmutiri@kau.edu.sa; 5 Department of Prosthetic Dental Sciences, College of Dentistry, Prince Sattam Bin AbdulAziz University, Alkharj 11942, Saudi Arabia; dr_kmq@hotmail.com; 6 Department of Prosthetic Dental Science, College of Dentistry, King Saud University, P.O. Box 60169, Riyadh 11545, Saudi Arabia; tajabbar@ksu.edu.sa; 7 Research Chair for Biological Research in Dental Health, College of Dentistry, King Saud University, Riyadh 11545, Saudi Arabia

**Keywords:** dental caries, anthropometry, body height, body weight, children

## Abstract

*Aim*: This study aimed to investigate an association between dental caries status and anthropometric measures in primary school children. *Methods and Materials*: An analytical cross-sectional study (*n* = 376) was conducted among primary school children (age range = 6–9 years) registered in private schools. Non-clinical data was gathered from parents of participating children through a self-administered structured questionnaire as well as from the children through an interviewer-administered questionnaire. Clinical data included the examination of dental caries using dmft/DMFT index and anthropometric measures including calculated z-scores of height-for-age (HAZ), weight-for-age (WAZ), BMI-for-age (BAZ), and physical examination. Inferential statistics included Kruskal Wallis and linear regression for univariate and multivariate analysis respectively. *Results*: The proportion of dental caries in primary and secondary dentition was 67.6% and 8.2% respectively. A significant association was observed between dental caries status and HAZ, WAZ, and BAZ (*p* < 0.001). An inverse relation was found between low, medium, and high dental caries categories and anthropometric measures. *Conclusions*: In the primary dentition, dental caries were significantly and inversely related to weight-for-age, height-for-age, and BMI-for-age. Hence, it can be concluded that among the low-income population dental caries is associated with lower anthropometric outcomes in children and therefore caries management should be considered an approach impacting overall health and quality of life.

## 1. Introduction

Dental caries in childhood is the 2nd most common and prevalent condition adding up to the global burden of chronic diseases [1]. Findings of Centers for Disease Control and Prevention (CDC) have revealed that 50% of schoolchildren aged 6–11 exhibit dental caries. Its untreated form is not only a financial burden but may have adverse effects on quality of life [2]. Studies have shown that school children having carious teeth may affect their weight and height. Dental caries, on one hand, maybe associated with obesity or on the other hand with underweight/stunting; both of these are forms of malnutrition. Both, obesity and underweight in children have deteriorating consequences on child’s health, growth, and quality of life. Impaired physical development due to underweight and stunting leads to alteration in mental health resulting in lower IQ, causing lack of educational achievement [3].

Numerous mechanisms have suggested a link between dental caries and the poor development of a child. Firstly, there is a direct influence of untreated tooth decay which leads to pain and inflammation-causing functional limitations resulting in poor caloric intake. Secondly, the indirect impact of caries causes changes in immune, endocrine, and metabolic responses [4]. Dental pain and pulp infections impact endocrine responses disturbing the slow sleep wave leading to imbalances in growth hormones altering the height and weight [4,5].

Changes in growth all over the world have been measured by anthropometry it is taken as an important indicator of a child’s growth and development. WHO growth references are available for screening and monitoring a child’s development measured in terms of Z-score or standard deviation (SD). Z-score is recommended in children as the value for one child might differ from other with respect to age and gender [6,7,8]. It also facilitates comparison with the reference population by determining exact standard deviation units from the reference distribution, hence provides more precision by identifying fixed points in the distribution in children. In adults overall height, weight and BMI alone is a sufficient indicator of anthropometry. Anthropometric measurements like weight-for-age (WAZ), height-for-age (HAZ), and BMI-for-age (BAZ) are used as a proxy of a child’s nutritional status and growth [6,7,8]. Many studies have reported the relationship between dental caries and child growth parameters providing inconclusive and inconsistent results [4,9]. Most of the studies conducted previously were based on children in Western countries and have reported inconsistent and contradictory results which served as a basis for this study [10]. The association of child growth with caries status however differs in countries with low and high average population income [11,12]. High-income countries exhibit an association between an increase in caries levels and greater BMI whereas in low-income countries, dental caries exhibits an inverse linear relationship. Besides, the management of dental caries is usually followed by a subsequent increase in weight [13].

Therefore this study aimed to assess the dental caries status of primary school children and its association with their anthropometric measures calculated as z-scores of height-for-age (HAZ), weight-for-age (WAZ), and BMI-for-age (BAZ).

## 2. Materials and Methods

### 2.1. Ethical Statement

Ethical approval was acquired from Institutional Review Board (IRB-918/DUHS/Approval/2017/141) and the study methodology was approved by the Board of Advance Studies and Research of University (DUHS/BASR/2017/-73). Permission for data collection was sought by the school administrations and consent was taken from parents of participating children. All participants were allowed to drop out at any point of study.

### 2.2. Study Setting and Participants

An analytical cross-sectional study was conducted from July 2018 to July 2019. Study participants were selected from private primary schools. A minimum sample of 342 was calculated using a proportion of underweight children with dental caries as 33.4%, at 95% confidence interval, 5% level of significance, and 5% margin of error [14]. The sample size was increased to 410 by adding 20% to overcome the missing data. Seven schools were selected based on convenience sampling whereas; consecutive sampling technique was applied for the selection of study participants. The Inclusion criteria were 1–4 grade students bearing ages between 6–9 years. The reason for targeting this age group was that growth at this age is stable, pre-pubertal, ability to respond to questionnaire and primary teeth have got enough exposure to the oral environment. Exclusion criteria included children suffering from acute infections, fever, diarrhea, ≤7 days history of vomiting, anodontia, oligodontia or supernumerary teeth, insufficient mouth opening, history of parasitic infestations, absence on the day of questionnaire distribution, or returning unfilled forms.

**Figure 1:**
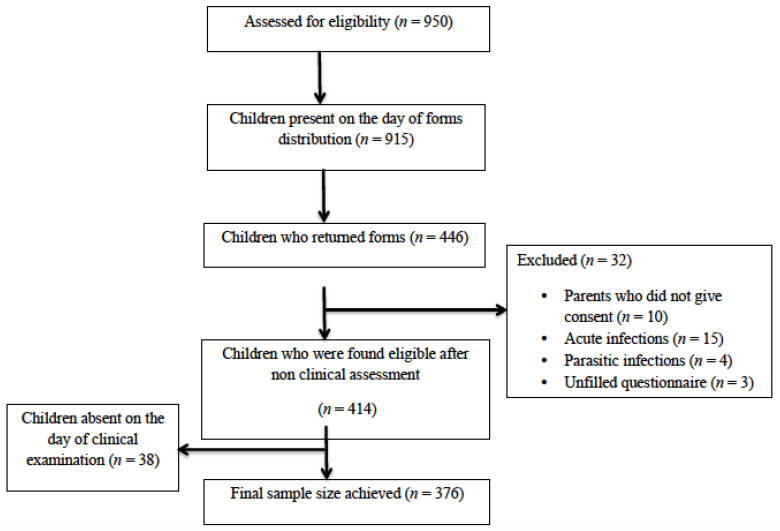
Flow Diagram.

Data collection proceeded after the selection of study participants and obtaining informed consent (Figure 1). Non-clinical data was gathered from parents as well as participating children through two different questionnaires. First was the parental questionnaire in which parents were asked to fill a self-administered structured questionnaire which was sent to them in child’s daily diary, this questionnaire was based on sociodemographics, child health variables, child’s appetite, and history of dental pain. Parents were given one week to complete and return the questionnaire and consent form. Non-clinical data obtained from the parental questionnaire was gathered to assess the inclusion and exclusion criteria of study participants. Another was the child/parent questionnaire, shortlisted children (from the questionnaire sent to parents previously) were interviewed. These questions were based on the frequency of sugar consumption by a child on a daily and weekly basis, and the questions were asked from the child in the presence of the parent/guardian by the interviewer. Following this, clinical data were collected from children, which comprised of anthropometric examination and dental examination by a single investigator (WA).

### 2.3. Questionnaire for Parents

The questionnaire was designed in English but was also translated into regional language. Parental Questionnaire was adapted from a previous study with some additional questions like the history of low birth weight and history of premature birth under “general information about the child” as these were the shortcomings of the previous study [15]. The questionnaire was divided into four sections: Socio-demographics of participants: variables like name of the child, date of birth, class, the gender of the child, contact number, occupation and education of parents, total monthly income and total number of family members. General information about child: history of gestational weeks at birth, birth weight, past hospitalization, chronic illness, infections in past 6 months of >7 days, anemia, medication, dental pain, food supplements, parasitic infestations, diarrhea, vomiting and fever in last one week. Child’s appetite: current status of child’s appetite, changes in appetite. History of child’s dental pain: difficulty in sleeping, eating, speaking, learning and absence from school due to dental pain in last three months. Questionnaire for children was based on history of dietary sugars and frequency of brushing.

### 2.4. Dental Caries Examination

It was performed using WHO Oral Health Survey Methods-2013 [16]. Scores for primary and permanent teeth were recorded using dmft and DMFT index respectively. Decay was considered when there was an obvious cavitation level as per WHO criteria and coding.

### 2.5. Anthropometric Measurements

Anthropometric Indicators Measurement Guide was used for measuring height, weight, and BMI on a separate form [17]. Weight was measured using a portable electronic weighing machine to the nearest 0.1 kg whereas height was recorded using stadio-meter to the nearest 0.5 kg following the standard protocol. Three readings were taken for each unless there is a variation of more than 100 g or 0.5 cm in that case fourth reading was taken. The median of these readings was recorded.

### 2.6. Confounders

Sociodemographic variables like: (i) age of the participant, gender, parental occupation, parental education, family income, number of family members (ii) child health variables like gestational weeks at birth, birth weight, past major hospitalization, chronic illness, infection in past 6 months, previous advise for the blood test, physical examination of anemia, history of medicines, history of dental pain, food supplements, average hours of sleep (iii) variables about child’s appetite like changes in appetite, sweets consumption (iv) dental variables: difficulty in eating, sleeping, speaking, learning, absence from school were considered confoundred of the present study.

Physical examination of anemia was performed in the lower palpebral conjunctiva, mucosal lining, palmer creases, nail beds, and general body skin. Anemia was marked as being present or absent only.

### 2.7. Statistical Analysis

Dependent variables included HAZ, WAZ, and BAZ scores. Independent variables include dmft/DMFT, socio-demographic variables, child health variables, variables on child appetite and variables on dental pain. Z-score calculation was done by using formula difference between an individual’s value and median value of the reference population with respect to particular ager or gender. Weight, height and BMI was recorded as continuous variables, for the purpose of easy z-scores calculation was done automatically through WHO anthroplus software using cutoff WAZ < −2 SD indicating underweight, HAZ < −2 indicating stunting, BAZ < −2 indicating underweight for a specific age and gender, BMI > +1 SD indicated overweight and BMI > +2 indicated obesity [15,17].

DMFT and dmft were recorded as continuous variables based on WHO criteria and coding but for the purpose of analysis they were converted into categories. Since no standardized classification for caries severity is available for the mentioned age group, therefore, two classifications were developed which were used to check the trend effect. The first was dmft classification in which the data set was divided into three comparable groups that are low, medium, and high caries groups known as tertiles (Tertiles classification): dmft = 0–1, dmft = 2–3, and dmft = ≥4. Second dmft classification had 4 categories (Caries-free group and low, medium, high caries group) in this classification children with no caries were assigned a separate group whereas children with caries were again divided in three comparable groups, that is dmft = 0 (no caries), dmft = 1–2 (low caries group), dmft = 3 (moderate caries group) and dmft = ≥4 (high caries group). This method of classifying dental caries as per severity has been previously used in some studies [11,15]. These two classifications will facilitate a comparison between low, medium, and high caries groups as well as no caries and caries groups to attain a true picture of this relationship. Data were analyzed using statistical program for social sciences (SPSS v.21, IBM, New York, NY, USA). Descriptive stats like mean and SD were recorded for continuous variables whereas frequencies and percentages were obtained for categorical variables.

Since, data was not following normal distribution (*p* < 0.05), Kruskal-Wallis test was applied to look into the relationship between dental caries scores (dmft/ DMFT) and cut-off, z-scores. A generalized linear model was applied for univariate and multivariate analysis. In univariate analysis *p*-value < 0.25 and in multivariable analysis *p*-value < 0.05 was considered statistically significant.

## 3. Results

Initially, 950 students, enrolled in selected schools were assessed for eligibility. Nine hundred and fifteen students received the forms, out of which 446 returned them. After the application of inclusion and exclusion criteria, 414 were selected and the final sample achieved was 376 (Figure 1). Among participants, gender distribution was nearly equal with 187 (49.7%) boys and 189 (50.3%) girls. The mean age for all children was 8.03 ± 1.03 years. There was no difference between mean age of girls and boys.

Socio-demographics of study participants are presented in Table 1. Male to female participants’ ratio was almost equal in this study. Father of most of the participants were privately employed, majority had their mother as housewives. Educational level of parents showed that majority of both parents had attained a university degree.

Table 2 shows dental caries scores of study participants. In primary teeth mean scores were as follows: decayed teeth (dt) 1.89 ± 1.92, missing teeth (mt) 0.01 ± 0.11, filled teeth (ft) 0.03 ± 0.18 whereas total mean dmft was 1.93 ± 1.97. Scores show that in permanent dentition mean DMFT was recorded as 0.09 ± 0.32.

A majority of the participants 358 (95.25), 355 (94.4%), 352 (93.6%), 332 (88.3%), 301 (80.1%), gave no history of absence from school, learning difficulties, speaking difficulty, sleeping difficulty and eating difficulties in past three month respectively.

In primary dentition dental caries prevalence was 67.6% whereas only 32.4% children were free of dental caries. In permanent dentition dental caries was 8.2%, whereas 91.8% were free of dental caries. Mean WAZ, HAZ and BAZ was found to be −0.4197, −0.1692, −0.4698 respectively, whereas median was −0.5500, −0.2150, −0.6000 respectively. Table 3 shows mean differences between dental caries and anthropometric outcomes (*n* = 376).

In Tertile’s classification a significant and inverse relationship was found between dmft categories (low, medium and high dental caries categories) and anthropometric measurements (*p* = <0.001). Also, a decline in anthropometric outcomes was seen and a negative linear trend was observed between dental caries categories and HAZ, WAZ and BAZ. A significant relationship was found between caries free, caries categories and HAZ, WAZ and BAZ (*p* = <0.001). No caries group showed positive values for HAZ, WAZ and BAZ. With the increase in dental caries score, a decline in anthropometric outcomes was observed.

Table 4 shows correlations between dental caries and height, weight, and BMI. The mean dmft was found strongly and inversely correlated with the HAZ (r = −0.649), WAZ (r = −0.766), and BAZ (r = −0.641) which was highly significant (*p* < 0.001). However, weaker co-relation was seen between the mean DMFT and HAZ, WAZ and BAZ.

The unadjusted generalized linear model showed that WAZ, HAZ, BAZ was significantly associated with dental caries and some of the confounders like socio-demographic variables, child health variables, child appetite and dental pain. After adjustments, the results of multivariate analysis showed that WAZ, HAZ and BAZ scores were still significantly associated with dmft categories and other variables (*p* < 0.001) Table 5.

## 4. Discussion

In low-income countries, child health is far behind the international goals affecting educational levels, general development, and wellbeing of children [18,19]. It was hypothesized that dental caries was significantly associated with WAZ, HAZ, and BAZ. The findings of the present study accept this hypothesis. In this study, dental caries was significantly and inversely associated with anthropometric measurements like WAZ, HAZ, and BAZ even after adjustment of confounding variables like socio-demographic variables, child health variables, child appetite, and dental pain.

In the present study, 6–9-year-old students attending private schools were included. Growth among children in this age group is quite stable (pre-pubertal) with the ability to respond to a questionnaire. It was seen that dental caries prevalence in primary dentition was higher in the present study (67.6%) in comparison to the reports of CDC which was 45.2% for this age group [2,20]. However, when the results were compared to the study conducted by Alkarimi et al., the prevalence of dental caries in primary dentition is lower than dental caries in primary school children, which was 87.1% [15]. The reason behind the low prevalence of dental caries among our study participants could be since we included children from middle-income families only, whereas in other studies students from all socioeconomic backgrounds were included. In contrast to the results of primary dentition in the current study, caries prevalence in permanent dentition was low (8.2%). This is attributed to the fact that the study population was aged between 6–9 years, and has not been exposed to the oral environment for longer periods, resulting in lower caries experience. Hence, this finding (caries in permanent dentition) is in line with the global goal for oral health by WHO, which was to increase the number of caries-free children by 2020 [21].

Despite, a quarter of parents reported that children had experienced dental pain only once in their lifetime, the prevalence of untreated caries remained high in the study participants. This also suggests that dental pain and caries is usually neglected in low-income countries. Another key finding of the current study is frequent snacking and intake of sugary foods by the participants.

Evidence between the relationship of dental caries and anthropometric measures is inconclusive and inconsistent as stated in the literature. In the present study, a significant association was seen between dental caries experience in school children and anthropometric measurements like WAZ, HAZ, and BAZ. Studies on assessment of nutritional status in young children with severely decayed teeth suggested that dental caries might be a risk factor for malnutrition [22]. This significant association between dental caries and anthropometric outcomes found in the current study is in relation to other reported studies [5,11,18,23,24,25].

An inverse linear trend was detected between levels of dental caries and anthropometric outcomes. This trend remained statistically significant even after adjustment for sociodemographic and other confounding variables. Hence, it can be stated that the results of the present study partially agree with one wing of the pattern suggested by Hooley et al. and Li et al. hypothesis. Since, they explained that dental caries and BMI might be related to each other in a U-shape non-linear pattern [9,26].

Studies performed in other developing countries (low and middle income) reported an inverse relationship with dental caries and anthropometric measurement [15,27]. A study by Oliveria et al. studied the relationship between primary teeth and height, also it was reported that children who had a high level of caries were significantly more likely to be shorter in height in comparison to caries-free children who had low levels of dental caries [28]. Similarly, Nbuab found children with dental caries exhibited greater odds of being underweight and stunted after a two-year follow-up. Moreover, underweight and stunting were significantly associated with the late eruption in children [23]. It was seen that with the increase in dental caries scores the scores for HAZ, WAZ and BAZ kept declining linearly. These findings are in agreement with the present study since we have found an inverse relationship between dental caries categories and all the anthropometric measurements.

On the contrary, a recent study conducted on Canadian preschool children with ECC found a significant relationship with greater BMI when compared to caries-free children [27]. Similar findings were reported in European and US studies which drew the conclusion that this connection might be because risk factors are the same for dental caries and increased BMI including excessive intake of sugary food, and frequent snacking [27,29]. From the above-mentioned studies, it can be concluded that in high-income countries a relationship exists between dental caries and overweight.

Some randomized controlled trials on children with severe untreated caries exhibited improvements in weight after dental management [13,30]. In this study parents of 4.8% of children reported that their children got absent from school due to toothache in the past three months. Absence from school is another indicator of analyzing the role of dental caries on academic performance [31] Reports have shown that in the South Asian Region prevalence of underweight is high, As a result, when such children grow up they lead to a less productive society, intensifying the magnitude of unemployment and poverty in the country. The above-mentioned scenario must be considered as alarming as we have found a relationship between dental caries and growth which might be an indicator for a health problem in children which deserves attention [32].

Some limitations of this study include the use of dmft/DMFT index as a dental caries assessment tool that identifies caries at its initial stage and is unable to measure caries severity. This may influence the detection of functional limitations in children. Also, a cause-and-effect relationship is not established in the present study due to cross-sectional design. Therefore, further randomized controlled trials investigating the long-term influence of caries and its extent among mixed dentition children associated with weight, height, and BMI are recommended.

## 5. Conclusions

This study shows that in mixed dentition (6–9 years), dental caries in primary dentition are prevalent in contrast to permanent dentition. It was observed that in primary dentition, dental caries was significantly and inversely related to weight-for-age, height-for-age, and BMI-for-age. Hence, it can be concluded that among the low-income population dental caries is associated with lower anthropometric outcomes in children and therefore caries management should be considered an approach impacting overall health and quality of life.

## Figures and Tables

**Table 1:** Socio-demographics characteristics of study participants (*n* = 376).

Variables		*n*	%
**Gender**			
	Male	187	49.7%
	Female	189	50.3%
**Father’s occupation**			
	Government officer	66	17.6%
	Private Job	250	66.5%
	Retired	11	2.9%
	Unemployed	7	1.9%
	Others	42	11.2%
**Mother’s occupation**			
	Working	63	16.8%
	House wife	313	83.2%
**Father’s education**			
	No formal primary schooling	6	1.6%
	Primary schooling	16	4.3%
	Secondary schooling	45	12.0%
	Higher school level	51	13.6%
	University degree	189	50.3%
	Above bachelor degree	69	18.4%
**Mother’s education**			
	No formal primary schooling	16	4.3%
	Primary schooling	21	5.6%
	Secondary schooling	55	14.6%
	Higher school level	90	23.9%
	University degree	161	42.8%
	Above bachelor degree	33	8.8%

**Table 2:** Dental caries status of the child (*n* = 376).

		Mean ± SD	Median	Min-Max
**Primary teeth**				
	decayed teeth (dt)	1.89 ± 1.92	1.0	0–9
	Missing teeth (mt)	0.01 ± 0.11	0.0	0–1
	Filled teeth (ft)	0.03 ± 0.18	0.0	0–1
	Total dmft score	1.93 ± 1.97	1.0	0–9
**Permanent teeth**				
	Decayed teeth (DT)	0.09 ± 0.32	0.0	0–3
	Missing teeth (MT)	0.0 ± 0.0	0.0	0
	Filled teeth (FT)	0.0 ± 0.0	0.0	0
	Total DMFT score	0.09 ± 0.32	0.0	0–3

SD: standard deviation.

**Table 3:** Mean differences in anthropometric outcomes according to dmft (*n* = 376).

		HAZ		WAZ		BAZ	
Dental Caries		Z-Score (WHO)		Z-Score (WHO)		Z-Score (WHO)	
	*n* (%)	Mean ± SD	*p*-Value *	Mean ± SD	*p*-Value *	Mean ± SD	*p*-Value *
**Tertile classification**							
dmft = 0–1 caries	194 (51.6%)	0.37 ± 1.05	<0.001	0.34 ± 1.16	<0.001	0.18 ± 1.12	<0.001
dmft = 2–3 caries	99 (26.3%)	−0.49 ± 0.76		−0.79 ± 0.83		−0.79 ± 0.97	
dmft = ≥4 caries	83 (22.1%)	−1.05 ± 0.83		−1.76 ± 0.85		−1.61 ± 0.92	
**Caries free and tertile classification**							
dmft = 0 (no caries)	122 (32.4%)	0.62 ± 1.05	<0.001	0.64 ± 1.17	<0.001	0.38± 1.15	<0.001
dmft = 1–2 caries	126 (33.5%)	−0.10 ± 0.81		−0.31 ± 0.88		−0.38 ± 1.00	
dmft = 3 caries	45 (12.0%)	−0.85 ± 0.72		−1.11 ± 0.84		−0.92 ± 0.98	
dmft = ≥4 caries	83 (22.1%)	−1.05 ± 0.83		−1.7 ± 0.85		−1.6 ± 0.92	

WHO = World Health Organization; SD = Standard deviation; dmft = decayed missing filled primary teeth; * *p*-value calculated using Kruskal Wallis test.

**Table 4:** Correlations between dental caries and height, weight and BMI.

		HAZ	WAZ	BAZ
dmft	r	−0.649	−0.766	−0.641
	*p*-value	<0.001	<0.001	<0.001
DMFT	r	−0.081	−0.085	−0.06
	*p*-value	0.111	0.099	0.244

r: Spearman’s correlation coefficient.

**Table 5:** Univariate and multivariate analysis (generalized linear model).

	Univariate Analysis (*p*-Value)			Multivariate Analysis (*p*-Value)		
Variables	WAZ	HAZ	BAZ	WAZ	HAZ	BAZ
Dental caries						
Caries free group dmft =0 (ref)						
dmft = 1–2	<0.001	<0.001	<0.001	<0.001	<0.001	<0.001
dmft = 3	<0.001	<0.001	<0.001	<0.001	<0.001	<0.001
dmft >= 4	<0.001	<0.001	<0.001	<0.001	<0.00	<0.001
Age	0.983	0.091 *	0.266	-----	0.149	-----
Gender of child	0.331	0.247 *	0.499	-----	0.326	------
Father’s occupation	0.412	0.980	0.237 *	-----	0.047 *	0.505
Mother’s occupation	0.447	0.223 *	0.547	-----	0.134	-----
Father’s education	0.007 *	0.004 *	0.039 *	0.016 *	<0.001 *	0.293
Mother’s education	0.881	0.688	0.793	------	----	----
Income	0.90	0.68	0.272	-----	----	----
Family members	0.131 *	0.531	0.023 *	0.807	----	0.423
Gestational weeks at birth	0.519	0.568	0.380	-----	-----	-----
Birth weight	0.125 *	0.115 *	0.161 *	0.090 *	0.763	0.357
Past major hospitalization	0.693	0.284	0.786	0.146	0.253	0.536
Chronic illness	0.857	0.886	0.930	----	----	----
Infection in past 6 months	0.969	0.857	0.572	----	----	----
Previous advice by the doctor for a blood test	0.050 *	0.137 *	0.153 *	0.146	0.253	0.536
Clinical examination of anemia	<0.001 *	0.002 *	0.002 *	0.009 *	0.010 *	0.100
History of medication	0.221 *	0.094 *	0.187 *	0.104	0.011 *	0.273
History of dental pain	0.488	0.275	0.329	----	-----	-----
Food supplements intake	0.020 *	0.137 *	0.203 *	0.469	0.704	0.829
Average hours of sleep	0.818	0.268	0.880	----	----	----
Recent changes in child’s appetite	0.614	0.892	0.295	----	----	----
Difficulty in eating/sleeping/speaking learning	0.007 *	0.072 *	0.002 *	0.469	0.704	0.829
Absent from school due to toothache	0.068 *	0.957	0.577	0.071	---	---
Sweets and snack consumption	0.009 *	0.021 *	0.018 *	0.018 *	0.383	0.051

HAZ = High-for-age z-score; WAZ = Weigh-for-age z-score; BAZ = BMI-for-age z-score. * statistical significance.

## Data Availability

Study data is available form the corresponding author on individual request.

## References

[1] Rouxel P., Chandola T. (2018). Socioeconomic and ethnic inequalities in oral health among children and adolescents living in England, Wales and Northern Ireland. Community Dent. Oral Epidemiol..

[2] CDC Prevalence of Total and Untreated Dental Caries Among Youth: United States, 2015–2016. https://www.cdc.gov/nchs/data/databriefs/db307.pdf.

[3] Schmidt A.L., Strack M.H., Conde S.R. (2018). Relationship between food consumption, nutritional status and school performance. J. Hum. Growth Dev..

[4] Paisi M., Kay E., Bennett C., Kaimi I., Witton R., Nelder R., Lapthorne D. (2019). Body mass index and dental caries in young people: A systematic review. BMC pediatrics..

[5] Shen A., Bernabé E., Sabbah W. (2020). Severe dental caries is associated with incidence of thinness and overweight among preschool Chinese children. Acta Odontol. Scand..

[6] Tosi F. (2020). The Anthropometric Reference Data. Design for Ergonomics.

[7] World Health Organization (WHO) (2019). Nutrition Landscape Information System (NLIS) Country Profile Indicators: Interpretation Guide.

[8] Fernández L., Rubini A., Soriano J.M., Aldás-Manzano J., Blesa J. (2020). Anthropometric assessment of Nepali children institutionalized in orphanages. Children.

[9] Li L.W., Wong H.M., Peng S.M., McGrath C.P. (2015). Anthropometric measurements and dental caries in children: A systematic review of longitudinal studies. Adv. Nutr..

[10] Carson S.J. (2018). No consistent association found between dental caries and body mass index in children. Evid. Based Dent..

[11] Mishu M.P., Tsakos G., Heilmann A., Watt R.G. (2018). Dental caries and anthropometric measures in a sample of 5- to 9-year-old children in Dhaka, Bangladesh. Community Dent. Oral Epidemiol..

[12] Oyapero A., Adenaike A., Edomwonyi A., Adeniyi A., Olatosi O. (2020). Association between dental caries, odontogenic infections, oral hygiene status and anthropometric measurements of children in Lagos, Nigeria. Braz. J. Oral Sci..

[13] Duijster D., Sheiham A., Hobdell M.H., Itchon G., Monse B. (2013). Associations between oral health-related impacts and rate of weight gain after extraction of pulpally involved teeth in underweight preschool Filipino children. BMC Public Health.

[14] Mishu M.P., Watt R., Tsakos G., Heilmann A. (2016). Associations between dental caries and BMI among 5–9 year old Bangladeshi childrenMasuma Pervin Mishu. European J. Public Health.

[15] Alkarimi H.A., Watt R.G., Pikhart H., Sheiham A., Tsakos G.J.P. (2014). Dental caries and growth in school-age children. Pediatrics.

[16] Petersen, Erik P., Baez, Ramon J., World Health Organization (2013). Oral Health Surveys: Basic Methods.

[17] Cogill B. (2003). Anthropometric Indicators Measurement Guide. Food and Nutrition Technical Assistance Project.

[18] Freire M.D., Corrêa-Faria P., Costa L.R. (2018). Effect of dental pain and caries on the quality of life of Brazilian preschool children. Revista de Saude Publica.

[19] Chala S., El Aidouni M., Abouqal R., Abdallaoui F. (2017). U-shaped association between untreated caries and body mass index in adults at Rabat dental University hospital, Morocco: Cross sectional study. BMC Res. Notes.

[20] Umer M.F., Farooq U., Shabbir A., Zofeen S., Mujtaba H., Tahir M. (2016). Prevalence and associated factors of dental caries, gingivitis, and calculus deposits in school children of sargodha district, pakistan. J. Ayub Med. Coll. Abbottabad.

[21] Hunter M. (2018). Oral Health 2020: Recommendations to Help Ohio Meet Healthy People 2020 Goals Based on States’ Oral Health Plans. Master’s Thesis.

[22] De Onis M., Garza C., Onyango A., Rolland-Cachera M. (2009). WHO growth standards for infants and young children. Archives de Pediatrie Organe Officiel Societe Francaise Pediatrie.

[23] Dimaisip-Nabuab J., Duijster D., Benzian H., Heinrich-Weltzien R., Homsavath A., Monse B., Sithan H., Stauf N., Susilawati S., Kromeyer-Hauschild K. (2018). Nutritional status, dental caries and tooth eruption in children: A longitudinal study in Cambodia, Indonesia and Lao PDR. BMC Pediatrics.

[24] Davidson K., Schroth R.J., Levi J.A., Yaffe A.B., Mittermuller B.A., Sellers E.A. (2016). Higher body mass index associated with severe early childhood caries. BMC Pediatrics.

[25] Koksal E., Tekçiçek M., Yalcın S.S., Tuğrul B., Yalçın S., Pekcan G. (2011). Association between anthropometric measurements and dental caries in Turkish school children. Cent. Eur. J. Public Health..

[26] Hooley M., Skouteris H., Millar L. (2012). The relationship between childhood weight, dental caries and eating practices in children aged 4–8 years in Australia, 2004–2008. Pediatric Obes..

[27] Shim S.H., Han D.H., Khang Y.H. (2018). Association between dental caries and delayed growth in Korean children. Caries Res..

[28] Oliveira L.B., Sheiham A., Bönecker M. (2008). Exploring the association of dental caries with social factors and nutritional status in Brazilian preschool children. Eur. J. Oral Sci..

[29] Hayden C., Bowler J.O., Chambers S., Freeman R., Humphris G., Richards D., Cecil J.E. (2013). Obesity and dental caries in children: A systematic review and meta-analysis. Community Dent. Oral Epidemiol..

[30] Alkarimi H.A., Watt R.G., Pikhart H., Jawadi A.H., Sheiham A., Tsakos G. (2012). Impact of treating dental caries on schoolchildren’s anthropometric, dental, satisfaction and appetite outcomes: A randomized controlled trial. BMC Public Health.

[31] Neves É.T., Firmino R.T., de França Perazzo M., Gomes M.C., Martins C.C., Paiva S.M., Granville-Garcia A.F. (2016). Absenteeism among preschool children due to oral problems. J. Public Health.

[32] Bhutta Z.A., Soofi S.B., Zaidi S.S.H., Habib A. (2011). Pakistan National Nutrition Survey.

